# The fossil record of the genus *Varanus* from the Southern Caucasus (Armenia, Georgia)

**DOI:** 10.7717/peerj.8322

**Published:** 2020-01-02

**Authors:** Davit Vasilyan, Maia Bukhsianidze

**Affiliations:** 1 JURASSICA Museum, Porrentruy, Switzerland; 2 Department of Geosciences, University of Fribourg, Fribourg, Switzerland; 3 Georgian National Museum, Tbilisi, Georgia

**Keywords:** *Varanus*, Late Miocene, Southern Caucasus, Armenia, Georgia, Palaeobiogeography, Palaeoclimate

## Abstract

The Southern Caucasus, with its special geographic position and complex topography, is a well-known biodiversity hotspot. However, the formation of this hotspot remains largely unstudied. To reveal this, a thorough study of the fossil record of the region is necessary. In the present paper, we describe for the first time fossil monitor lizards (*Varanus* sp.) from two late Miocene localities from the Southern Caucasus (Jradzor, Armenia and Tetri Udabno, Georgia). We suggest that both fossils belong to a small-sized monitor lizard, comparable to the present-day species found in Iran and the Middle East (e.g., Iraq, Saudi Arabian)—the most western part of the extant monitor lizards’ Eurasian distribution range. Our finds show that the genus had a broad distribution in the Eastern Paratethyan region during the late Miocene. In addition, we provide the probable temperature ranges for fossil localities.

## Introduction

The Southern Caucasus is characterized by diverse biomes ranging from humid subtropical evergreen forests to dry steppe with numerous endemic plant and animal species (Nakhutsrishvili et al., 2015). Biotic diversity has been shaped on one hand by the topography of the region with their characteristic weather zones and on another hand due to the geographic position of the region at the crossroad of Europe, Asia and Africa. Due to the limited number of palaeontological studies in the region, the evolutionary history of these unique ecosystems and endemic forms remains largely unknown.

Though the history of the flora and fauna—molluscs, mammals, of the region were under the main focus of earlier studies (e.g., Hakopyan, 1974; Bukhsianidze & Koiava, 2018), other groups, e.g., such as the insects, amphibians and reptiles received less attention. Studies on the Neogene continental ectothermic vertebrate of the area are mainly limited to the turtles (Chkhikvadze, 1983; Chkhikvadze, 1989), whereas amphibian, crocodilian and snake (Gabunia, 1964; Chkhikvadze, 1984) remains are very scarce, as a result of the lack of rich fossil accumulations or insufficient excavation/or exploration.

In comparison to other regions of Western Eurasia, such as Anatolia, Eastern or Central Europe, the fossil record of Southern Caucasus is extremely poorly studied. This hinders on one hand the ability to trace the evolution of the local fossil record and the roots of the present-day unique ecosystems and endemic forms, but on another hand, it makes impossible any palaeobiogeographic comparison of the Southern Caucasian record with the other regions of Eurasia. This aspect has a crucial importance considering the key geographic location of the region.

**Figure 1:**
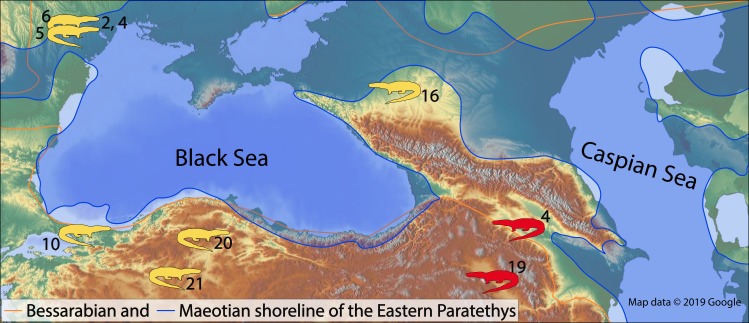
The map of the Eastern Paratethys with the known (in yellow) and herein described (in red) fossil occurrences of the genus *Varanus*. The numbers next to the fossil localities correspond to the locality numbers of the Table 1. Map data ©2019 Google.

In the present paper, we report the first fossil record of the genus *Varanus* from Southern Caucasus. We discuss and compare the fossil record of the genus with other similar age localities (Fig. 1, Table 1).

### Geological settings

#### Jradzor, Armenia

The studied material comes from the fossiliferous horizon JZ-3 of the Jradzor section in Central Armenia. The horizon is composed of clayey sand, representing an accumulation of weathering products from the surrounding volcanic rocks. Besides the monitor lizard remains, a rich vertebrate faunal assemblage has been discovered, which includes amphibians, reptilian, avian as well as mammalian remains (Vasilyan et al., 2018). The biochronology of small mammalian species from JZ-3 correlates the assemblage to the latest Miocene (latest MN13).

#### Tetri Udabno, Georgia

The fossils were found in the Tetri Udabno, one of the localities of the Udabno Site, Georgia, located in the most eastern part of the Udabno Syncline. So far, fossil vertebrates from this locality where known from the Shiraki Formation (Maeotian) (Bukhsianidze & Koiava, 2018). These fossil specimens come from the underlying Eldari Formation, continental deposits developed in the Middle Kura Basin, largely correlated with the Khersonian marine regional stage of the Eastern Paratethys. The monitor lizard remains were found in grey silty clays. So far, they are the only fossils found from the layer, yet, recently a large number of sporadically distributed vertebrate fossils on different stratigraphic levels in these deposits were revealed.

## Materials & Methods

The studied material represents partially articulated or disarticulated postcranial and jaw material found during the excavation/prospecting in 2017 in Georgia and 2018 in Armenia. The field work in Georgia has been provided by Maia Bukhsianidze, Georgian National Museum, whereas in Armenia it was undertaken by Davit Vasilyan, with support of the Institute of Geological Sciences, National Academy of Sciences of Republic of Armenia. The material has been photographed using a digital microscope (Leica DVM5000) and also a NIKON D610 camera. The terminology of Villa et al. (2018) has been used for the description of the fossil material. The measurements of the vertebrae follow those of Holmes et al. (2010) and Hocknull et al. (2009), which is indicated accordingly in the Table 2. The body length estimations have been calculated according to the ratio of snout-vent length (SVL)/vertebra length (Hocknull et al., 2009) introduced in Conrad, Balcarcel & Mehling (2012). The body size estimations have been calculated only based on the vertebrae for which the vertebral length Hocknull et al. (2009) can be measured. The material is stored in the palaeontological collections of the Institute of the Geological Sciences, National Academy of Sciences of Republic of Armenia, Yerevan, Armenia (IGS) and S. Janashia Museum of Georgia, Georgian National Museum, Tbilisi, Georgia (GNM1).

## Results

### Systematic palaeontology

**Table:** 

**Figure 2:**
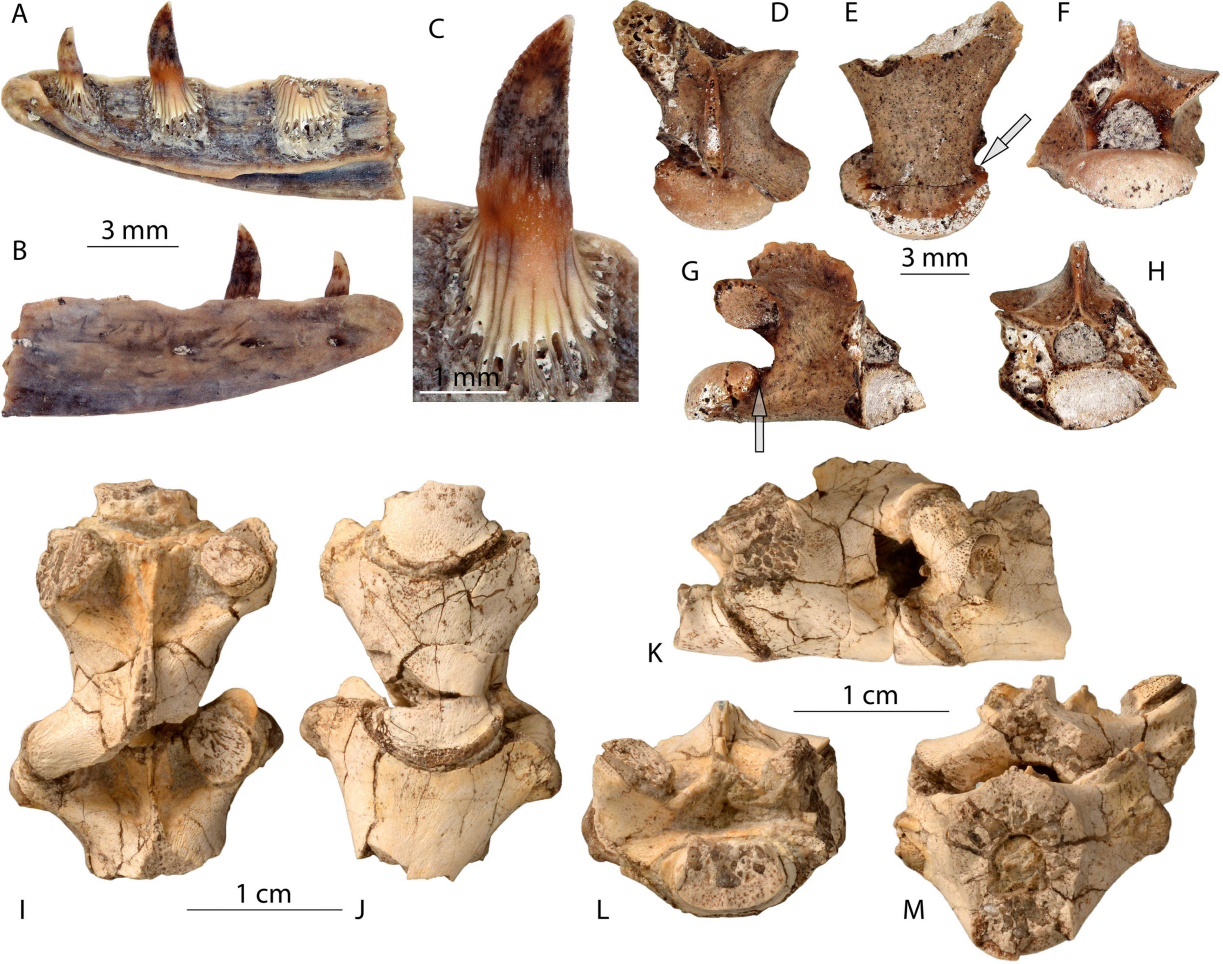
*Varanus* sp. from Armenia and Georgia. (A–C) right dentary, IGS JRD-18/12, and (D–H) trunk vertebra, IGS JRD-18/13 from locality Jradzor, horizon JZ-3, Armenia. (I–M) trunk vertebrae, GNM1 32-2013/1107-a - -f from locality Tetri Udabno, Georgia.

Clade Squamata Oppel, 1811
Clade Anguimorpha Fürbinger, 1900
Family Varanidae Gray, 1827
Genus *Varanus*Merrem, 1820
*Varanus* sp.
(Fig. 2)

### Material

One right dentary (IGS JRD-18/12) and one trunk vertebra (IGS JRD-18/13), Jradzor locality, horizon JZ-3, late Miocene, late Messinian, late MN13, Armenia. Ten trunk vertebrae (GNM1 32-2013/1107-a – -f) and three limb bones (GNM1 32-2013/1107-g – -i), locality Tetri Udabno, late Miocene, late Tortonian, Khersonian, Georgia.

**Table 1:** Late Miocene record of the genus *Varanus* from Western Eurasia.

	**Name**	**Country**	**Taxon**	**Age in Ma**		**Stage**	**Latitude**	**Longitude**	**Reference**
21	Çeştepe, Kazan Basin	Turkey	*Varanus* sp.	5–5.2		Zanclean	40.3252	32.6894	Sen, Delfino & Kazanci (2017)
20	Süleymanli	Turkey	*Varanus* sp.	5.3–7.1		Zanclean-Messinian	37.9000	36.8333	Böhme & Ilg (2003)
19	Jradzor-3 (JZ-3)	Armenia	*Varanus* sp.	5.3–6		Messinian			This study
18	Brisighella Cava Monticino	Italy	*Varanus* sp.	5.33–6		Messinian	44.2167	11.7667	Delfino (2002)
17	Polgárdi 5	Hungary	*Varanus* sp.	5.33–6.2		Messinian	47.0500	18.0300	Venczel (2006)
16	Solnechnodolsk	Russia	*Varanus* sp.	5.8–6.4		Messinian			Čerňanský, Syromyatnikova & Jablonski (2018)
15	Pollenzo section along Tanaro River, Verduno, Piedmont	Italy	*Varanus* sp.	5.42–5.55		Messinian	44.6858	7.9314	Colombero et al. (2014)
14	El Arquillo 1 (ARQ1)	Spain	*Varanus* sp.	6.23		Messinian	40.4000	−1.1000	Ivanov et al. (2018)
13	Samos 1	Greece	*Varanus marathonensis*	6.9–7.2		Messinian	37.8000	26.9000	Villa et al. (2018)
12	Pikermi near Athens	Greece	*Varanus marathonensis*	7.11–7.37		Messinian-Tortonian	38.0194	23.9917	Villa et al. (2018)
11	Kohfidisch	Austria	*Varanus* sp.	8.55–8.95		Tortonian	47.1667	16.3500	Tempfer (2005)
10	Küçükçekmece	Turkey	*Varanus* sp.	8.6–9.4	Khersonian	Tortonian	40.9833	28.7667	Vasilyan, Böhme & Prieto (2013)
9	Tetri Udabno	Georgia	*Varanus* sp.	7.6–9.6	Khersonian	Tortonian			This study
8	Cerro de los Batallones (Torrejón de Velasco), Madrid Basin	Spain	*Varanus marathonensis*	9–10		Tortonian	40.1794	−3.7246	Villa et al. (2018)
7	Ravin de la Pluie near Nea Messimvria, Axios Valley, 25 km W Thessaloniki	Greece	*Varanus* sp.	9.3		Tortonian	40.7530	22.7750	Georgalis et al. (2018)
6	Varnitza	Moldova	*Varanus* sp.	9.6–10.5	late Bessarabian	Tortonian	46.8641	29.4692	Lungu & Rzebik-Kowalska (2011)
5	Kalfa	Moldova	*Varanus* sp.	10.5–11.6	middle Bessarabian	Tortonian	46.9042	29.3753	Chkhikvadze & Lungu (1984)
4	Bushor 1	Moldova	*Varanus* sp.	10.5–11.6	middle Bessarabian	Tortonian	46.9225	28.2683	Lungu & Rzebik-Kowalska (2011)
3	Can Llobateres (Valles Penedes Basin. Barcelona)	Spain	*Varanus* sp.	9.64–9.74		Tortonian	41.5333	−2.1333	Ivanov et al. (2018)
2	Otovaska 1	Moldova	*Varanus* sp.	10.5–11.6	middle Bessarabian	Tortonian			Lungu & Rzebik-Kowalska (2011)
1	Petersbuch 18	Germany	*Varanus* sp.	11.5–12.5		Tortonian	48.9779	11.1909	Böhme (2003)

**Table 2:** Measurements of vertebrae and estimated body size of the studied specimens of *Varanus* sp. from Armenia and Georgia. Measurements follow * Holmes et al. (2010) and ** Hocknull et al. (2009). The estimated body size corresponds to the snout-vent length.

Collection numbers	Measurements (in mm)					Estimated body size (in mm) **
	min. centrum length*	max. centrum length*	condylar width*	precondylar constriction*	DVL**	
JRD-18/12	8	10.5	6.2	4.3 (69.3%)	8	290
GNM1 32-2013/1107-a1	–	–	–	6.2	–	–
GNM1 32-2013/1107-a2	12.6	16.1	10.45	6.5 (62.2%)	13	470
GNM1 32-2013/1107-a3	–	–	10.2	–	–	–
GNM132-2013/1107-b	–	–	∼9	–	–	–
GNM1 32-2013/1107-c1	–	–	–	7.4	–	–
GNM1 32-2013/1107-c2	–	–	10.3	–	–	–
GNM1 32-2013/1107-d1	–	–	–	6.6	–	–
GNM1 32-2013/1107-d2	–	–	10.5	–	–	–
GNM1 32-2013/1107-e	–	–	8.6	–	–	–
GNM1 32-2013/1107-f	–	–	–	6	–	–

### Description

The vertebrae from both localities belong to small-sized individuals. The estimated snout-vent length of the Armenian form (IGS JRD-18/13) is around 30 cm, whereas the Georgian one (GNM1 32-2013/1107-a2) is nearly 50 cm (Table 2).

An anterior portion of the right dentary is preserved. It shows three tooth positions. The dentary is slender and low. In labial view, the bone is flat and pierced by four small-sized dorsoventrally compressed mental foramina, which are arranged horizontally. In lingual view, the Meckelian groove is low and narrow. Below the third tooth, the groove opens medially and at the anterior part of the preserved fragment (the first and second tooth positions) the groove opens ventromedially. The symphyseal surface is oriented medioposteriorly. The teeth are linguolabially compressed and incline posteriorly. The first tooth measures two mm in height, the second four mm. The tooth base is broad and composed of a system of parallel-oriented striae which are directed to the tip of the tooth. Well-developed resorption pits are visible at the base of teeth, located between parallel-oriented striae. The transition from the tooth base to the tooth crown is narrow. The sharp distal and medial cutting edges of the tooth crown are serrated.

The studied vertebrae from both Jradzor and Tetri Udabno localities show the same morphology. The vertebrae are procoelous, and all of them originate from the trunk region. The vertebral centra are triangular in shape (anteriorly broad and posteriorly narrow). The condyle and cotyle are dorsoventrally strongly compressed. The dorsal margin of the cotyle projects over the ventral one. The surface of the condyle is smooth. The precondylar constriction is strongly pronounced. The anterior opening of the neural canal is round, whereas the posterior one has a flat ventral surface. In lateral view, the neural arch projects posterodorsally. Its most anterior portion is flat and forms a triangular surface in a depression. The dorsal surface of the neural arch possesses weakly- (Jradzor) or well-pronounced (Tetri Udabno) striae. The neural arch possesses relatively high neural crest. The pre- and postzygapophyses are bent laterodorsally.

An additional three bones with their partially-preserved diastemal and epiphyseal parts have been found from Tetri Udabno. Due to the preservation of the material, these remains cannot be referred to any bone. However, the fossil remains (vertebrae and long bones fragments) of *Varanus* sp. from Tetri Udabno belong most probably to one individual, because the bones are found together, have the same preservation, and the vertebrae are partially articulated. However, the long bone fragments from the same spot cannot be assigned with confidence to *Varanus* sp.

## Discussion

### Identification and comparison

The described fossil material shows characteristic features of the genus *Varanus*: (1) the presence of a system of well-pronounced and parallel-oriented laminae, as well as well-developed resorption pits at the base of the teeth (Kearney & Rieppel, 2006; Ivanov et al., 2018); (2) the vertebral centrum possesses a well-pronounced precondylar constriction (Smith, Bhullar & Holroyd, 2008; Conrad et al., 2011; Delfino et al., 2013); (3) the lateral and dorsal surfaces of the neural arch of the vertebrae display distinct and generally discontinuous striae (Smith, Bhullar & Holroyd, 2008).

Specific assignment of the studied material is impossible due to the lack of diagnostic characters and further skeletal elements relevant for identification. However, several differences from the already known Neogene forms of Europe can be mentioned. After the latest comprehensive revision of the European Neogene monitor lizard record, only two fossil species of the genus *Varanus* (*V. mokrensis* and *V. marathonensis*) are identified in Eurasia (Ivanov et al., 2018). At least four additional middle to late Miocene species have been described from the Eastern Paratethys area (Lungu, Zerova & Chkhikvadze, 1983; Zerova & Chkhikvadze, 1986) based on only isolated vertebrae. Unfortunately, all material is poorly documented and the taxonomic assignment needs a critical revision (Villa & Delfino, 2018). Thus, this record cannot be directly compared with Armenian and Georgian material. Ivanov et al. (2018) used the dimensions of the trunk vertebrae (the ratio of the condylar width to the precondylar constriction in %) to distinguish different species. Our studied material shows the smallest values of this ratio (Table 2, 69.3% in Jradzor and 62.2% in Tetri Udabno) vs. 81% in *V. mokrensis*, and 75–78% in *V. marathonesis* (Ivanov et al., 2018). Whether this difference could have a diagnostic significance to distinguish species, i.e., to assign the Southern Caucasian *Varanus* sp. to a separate species, needs to be tested further.

Taking into account the sizes of the bones, *Varanus* sp. from the Jradzor locality, Armenia could represent a subadult form. The characters such as tooth serration and degree of development of the striae on the vertebrae could be useful for the evaluation of the ontogenetic stage. However, as it has been shown (Smith, Bhullar & Holroyd, 2008; Hocknull et al., 2009), these characters have high intra- and interspecific variabilities, and it is rather difficult to use them.

It is interesting to note that most of the Asiatic varanids have developed trenchant and posteriorly directed teeth with serrated cutting edges (Ivanov et al., 2018) (similar to Armenian form). In addition to this, the most western present-day distribution of the genus *Varanus* is found in Iran (Anderson, 1999) and some parts of the Middle East (Pianka, King & King, 2004). Interestingly, here, it is known by two small-sized species *Varanus bengalensis* (SVL<750 mm) (Anderson, 1999) and *Varanus griseus* (SVL<860 mm) (Pianka, King & King, 2004), which, unfortunately, we lack for comparison.

## Conclusions

### Palaeobiogeographic and palaeoclimatic considerations

During the late Miocene, the genus *Varanus* was a common element of herpetofaunal assemblages in the west of the Eastern Paratethys region (Fig. 1, Table 1). Recent studies (e.g., Čerňanský, Syromyatnikova & Jablonski, 2018) and our finds strongly suggest their larger distribution covering regions west from the Black Sea. Monitor lizards are ectothermic and their palaeogeographic distribution depends largely on suitable climatic conditions. So, as suggested by Böhme (2003), the genus is characterized by the following climatic space: mean annual temperature 14.8–28.1 °C, mean warm month temperature of 13.9–26.1 °C, and mean cold month temperature of −3.9–19.4 °C. Comparable climatic conditions can be also expected in Jradzor and Tetri Udabno. During the late Miocene, prominent climatic changes have been documented both at the regional (Feurdean & Vasiliev, 2019) and global scale (Herbert et al., 2016). Undoubtedly, they shaped also the spatial and temporal distribution of all ectothermic vertebrates. Nevertheless, the herein documented new finds are the first records of monitor lizards in the region. More systematic fieldwork and studies are necessary to understand their fossil record in the region.

## Additional Information and Declarations

### Field Study Permissions

The following information was supplied relating to field study approvals (i.e., approving body and any reference numbers): The Institute of Geological Sciences approved the field work in Armenia. The fieldwork in Georgia has been provided by the leading national institution - Georgian National Museum.

### Data Availability

The following information was supplied regarding data availability: One right dentary (IGS JRD-18/12) and one trunk vertebra (IGS JRD-18/13), Jradzor locality, horizon JZ-3, late Miocene, late Messinian, late MN13, Armenia. Ten trunk vertebrae (GNM 32-2013/1107-a –-f) and three limb bones (GNM 32-2013/1107-g –-i), locality Tetri Udabnos Seri, late Miocene, late Tortonian, Khersonian, Georgia.
